# Dissociable age and memory relationships with hippocampal subfield volumes *in vivo*:Data from the Irish Longitudinal Study on Ageing (TILDA)

**DOI:** 10.1038/s41598-019-46481-5

**Published:** 2019-07-29

**Authors:** Daniel Carey, Hugh Nolan, Rose Anne Kenny, James Meaney

**Affiliations:** 1 The Irish Longitudinal Study on Ageing (TILDA), Trinity College Dublin, Dublin 2, Ireland; 2 Department of Medical Gerontology, Trinity College Dublin, Dublin 2, Ireland; 3 Department of Clinical Radiology, Trinity College Dublin, Dublin 2, Ireland; 4 Centre for Advanced Medical Imaging (CAMI), St. James’ Hospital, Dublin 8, Ireland

**Keywords:** Cognitive ageing, Hippocampus

## Abstract

The heterogeneous specialisation of hippocampal subfields across memory functions has been widely shown in animal models. Yet, few *in vivo* studies in humans have explored correspondence between hippocampal subfield anatomy and memory performance in ageing. Here, we used a well-validated automated MR segmentation protocol to measure hippocampal subfield volumes in 436 non-demented adults aged 50+. We explored relationships between hippocampal subfield volume and verbal episodic memory, as indexed by word list recall at immediate presentation and following delay. In separate multilevel models for each task, we tested linearity and non-linearity of associations between recall performance and subfield volume. Fully-adjusted models revealed that immediate and delayed recall were both associated with cubic fits with respect to volume of subfields CA1, CA2/3, CA4, molecular layer, and granule cell layer of dentate gyrus; moreover, these effects were partly dissociable from quadratic age trends, observed for subiculum, molecular layer, hippocampal tail, and CA1. Furthermore, analyses of semantic fluency data revealed little evidence of robust associations with hippocampal subfield volumes. Our results show that specific hippocampal subfields manifest associations with memory encoding and retrieval performance in non-demented older adults; these effects are partly dissociable from age-related atrophy, and from retrieval of well-consolidated semantic categories.

## Introduction

Hippocampus is among the most important brain structures involved in memory^1–3^, and is a critical site of pathogenesis in dementing illnesses such as Alzheimer’s Disease^4–7^. Decades of *ex vivo* human studies and *in vivo* animal studies have revealed anatomical and functional heterogeneity of the hippocampal subfields^8–12^. Yet, until recently, very few *in vivo* studies in humans had shown dissociable relationships between performance in different memory domains and hippocampal subfield anatomy or function^13–15^.

The role of the hippocampal subfields with respect to select domains of memory thus remains under explored. Recent work has shown that errors during real-world spatial navigation are negatively associated with hippocampal tail volume in mild cognitive impairment (MCI), but with Cornu Ammonis (CA) 3 volume in healthy controls^16^. Yet, despite this, there remains a limited understanding of the role of hippocampal subfields in other memory domains relied on in daily life, such as verbal episodic memory (e.g., recalling a grocery list) or semantic memory (e.g., retrieving familiar nouns) [but see^17^]. Verbal episodic and semantic memory have been shown to dissociate to hippocampal versus anterior temporal regions respectively^18–22^. However, much of our understanding of these dissociations is based on small and heterogeneous patient cohorts^19–21^. Little is known about these relationships in the context of healthy ageing; less still is known about the effects of age with respect to relationships between hippocampal subfields and specific memory domains, despite evidence of age-related variation in subfield anatomy^23–25^.

Recent advances in computational methods and atlases available for anatomical MRI have improved the reliability and efficiency with which histologically-validated hippocampal parcellations may be applied to *in vivo* datasets^9,26–29^. Spatially refined subfield parcellations have now been used in studies of clinical disorders^30,31^, and in one study of MCI and educational attainment^32^. Nevertheless, a pressing concern is the quantification of relationships between hippocampal subfields, memory domains, and age, particularly given projected worldwide growth in dementia prevalence in older adults^33^, and the key role of hippocampus in modulating memory^34^.

Here, we explored the role of hippocampal subfield anatomy with respect to two domains of memory, in healthy ageing. In a large, non-demented sample of community-dwelling older adults, we aimed to dissociate memory domains based on their expected patterning with hippocampal subfield volumes. We predicted relationships would emerge between verbal episodic memory (list learning and retrieval) and volumes of subfields CA1, CA2/3, CA4, and granule cell layer of dentate gyrus (GC-DG) – regions heavily implicated in encoding and retrieval processes^12,35^. In contrast, we expected that fluency in semantic memory (retrieval of familiar category names) would show little if any relationship with subfield volumes^19^. We appraised these relationships in tandem with age-related differences in subfield volumes, by assessing the robustness of effects related to memory alongside fits for cross-sectional age.

## Materials and Methods

### Design and participants

Details of TILDA’s design have been published previously^36,37^. Briefly, the study comprises a clustered stratified random sample of the population aged 50 and over living in the Republic of Ireland. At Wave 3 (2014), participants completed a computer assisted personal interview in their home (CAPI; N = 6,618; 85% response rate), and a physical health assessment with a trained research nurse at a health centre, or at home (N = 5,364; 82% response rate). Wave 3 participants who completed a health assessment were later invited to complete the MRI protocol. The study received ethical approval from the Research Ethics Committee, School of Medicine, Trinity College Dublin. All experimental procedures were performed in line with Trinity College Dublin School of Medicine guidelines and regulations for ethics in research involving human participants.

#### MRI sample

Initial recruitment prioritised participants aged 65 and over in order to limit attrition amongst the oldest old within the sample, with later recruitment targeting those aged 50–64 years. Participants provided voluntary informed consent before their scan appointment.

In total, 578 participants attended for MRI. T_1w_ datasets were acquired from 560 participants; 18 did not provide data (due to claustrophobia/nerves [n = 14], or MRI contraindication [n = 4]). Supplementary Fig. S1 indicates the exclusions made to the sample due to data quality issues (n = 51). We excluded 73 further participants based on physical and cognitive health criteria (Supplementary Fig. S1). A sample of 436 participants (see Table 1) were available for volumetric analyses (median age [IQR, age range]: 68 [65–73, 52-88]).

**Table 1:** Descriptive data for analysis sample.

Analysis sample (N = 436)	
Gender (n, %)	M: 207 (47.5) F: 229 (52.5)
Education (n, %)	Primary/None: 88 (20.2)
	Secondary: 170 (39.0)
	Third/Higher: 178 (40.8)
MoCA (median, IQR)	27 (25, 28)
MMSE (median, IQR)	29 (28, 30)
Immediate recall (median, IQR)	7 (6.5, 8.5)
Delayed recall (median, IQR)	7 (5, 8)
Animal naming (median, IQR)	19 (15.5, 23)
BMI (median, IQR)	27.7 (24.9, 30.5)
Timed up-and-go (median, IQR)	8.97 (8.0, 10.0)
Chair stands time^a^ (median, IQR)	13 (11, 15)
Falls (n, %)	Fall: 95 (21.8) No fall: 341 (78.2)
CHR - None, 1, 2+ (n, %)	None: 173 (39.7)
	1: 148 (33.9)
	2+: 115 (26.4)
CES-D (short form) quartiles (n^b^, %)	1^st^ (0): 142 (32.9)
	2^nd^ (1–2): 121 (28.0)
	3^rd^ (3–4): 72 (16.7)
	4^th^ (5+): 97 (22.4)
Cardiac disease history (n, %)	None: 369 (84.6) Yes: 67 (15.4)
Smoking history (n, %)	Never: 224 (51.4) Past/Current: 212 (48.6)

Continuous variables summarised as median ± inter-quartile range; categorical variables as %. MoCA - Montreal Cognitive Assessment. MMSE - Mini-mental State Examination. BMI - body mass index. Timed up-and-go: time taken to rise from chair, walk 3 metres, and return to seat. Chair stands: time taken to repeat five stands from seated position, without assistance of arms; ^a^data available for n = 395. Falls: any fall in the previous year. CHR: non-cardiac chronic conditions ordered count - none, one, two or more. CHR includes: cataracts, glaucoma, age-related macular degeneration, lung disease, asthma, arthritis, osteoporosis, cancer, substance abuse, ulcer, varicose ulcer, liver disease, thyroid condition, kidney disease, anaemia. CES-D quartiles: based on CES-D short form; ^b^data available for n = 432. Cardiac disease history: presence of at least one of abnormal heart rhythm, angina, cardiac arrest, or heart attack. Self-reported variables: gender, education, falls, CHR, cardiac disease history, smoking history; all other variables measured.

### Verbal episodic memory and semantic fluency assessment

Participants’ verbal episodic memory and semantic fluency performance were assessed by trained interviewers during the Wave 3 CAPI. Assessments comprised immediate and delayed verbal recall of word lists; semantic fluency was assessed by free naming of animals. All participants were fluent English speakers.

#### Immediate (IR) and delayed recall (DR)

IR and DR were tested during the cognitive module in the CAPI. One of four possible 10-item word lists was selected randomly by the CAPI computer (lists were the same as those validated by the Health and Retirement Study^38^; see Supplementary Methods). The list was then presented to the participant by audio recording, or by the interviewer reading aloud (in instances of difficulty hearing the recording; audibility was verified with a brief test recording played for the participant). Participants were instructed to listen to the entire list carefully (approx. rate: 1 word/2 s), and were then prompted to repeat as many of the presented words as possible within two minutes. The interviewer recorded the number of words recalled correctly. The test was then repeated at once using the same procedure. The participant’s IR score was calculated as the mean number of words recalled correctly across the first and second attempt. The CAPI then proceeded to the animal naming task (see below), followed by five further sections of questions (cardiac disease history; other chronic conditions; falls/fractures; pain; medical tests – duration ~25 mins). Following this, participants completed the DR test, which required them to repeat as many of the word list items from the IR test as possible; DR score was the number of words recalled correctly. IR and DR were weakly negatively correlated with age (IR: Spearman *ρ* = –0.27, *p* < 0.0001; DR: *ρ* = –0.25, *p* < 0.0001), and highly positively correlated with each other (*ρ* = 0.7, *p* < 0.0001).

#### Animal naming (AN)

Participants were instructed to name as many animals as possible in 60 seconds. Task timing was controlled by the CAPI computer. The interviewer recorded each word spoken by the participant, scoring as correct common nouns (including subordinate levels of categories, e.g., doe, stag), and as incorrect any repeated items or proper nouns. AN was weakly negatively correlated with age (*ρ* = –0.19, *p* < 0.0001), and weakly positively correlated with IR and DR (IR: *ρ* = 0.29, *p* < 0.0001; DR: *ρ* = 0.25, *p* < 0.0001).

### MRI protocol & T_1w_ acquisition

Scans were acquired at the National Centre for Advanced Medical Imaging (CAMI), St. James’ Hospital, Dublin, via 3 T Philip’s Achieva system with 32-channel head coil. A 3D Magnetisation-prepared Rapid Gradient Echo (MP-RAGE) sequence was used. FOV (mm): 240 × 218 × 162; 0.9 mm isotropic resolution; SENSE factor: 2; TR: 6.7 ms; TE: 3.1 ms; flip angle: 9°.

### Data inspection and hippocampal subfield reconstruction

All volumes were inspected for evidence of image artifact and presence of grey and white matter lesions by a trained operator blind to participant identity. Data for 33 participants were excluded due to motion artifact; 18 further datasets had one or more lesions present and were excluded (Supplementary Fig. S1). All T_1w_ image analyses were completed in FreeSurfer v.6.0^39–41^. We used the hippocampal subfields module within the FreeSurfer recon-all processing pipeline to segment hippocampus^26,39^. Details of these hippocampal segmentation routines have been published previously^26^. Briefly, the procedure employs a probabilistic atlas encoded in a tetrahedral mesh, and derived from manual segmentations using Bayesian techniques. Segmentation is posed as a Bayesian inference problem within a generative model, which spatially deforms atlas label prior probabilities; segmentations are achieved via Bayesian optimisation, based on the label prior probabilities and observed voxel intensities (see Supplementary Fig. S2). Recon-all procedures were run on a Linux computing cluster at the Trinity Centre for High Performance Computing. All hippocampal segmentations were inspected for error overlaid on the intensity normalised T_1w_ volumes by a trained operator blind to participant identity. All datasets had hippocampal segmentations that fell within expected tissue boundaries; none of the recon-all or subfield reconstruction procedures yielded any reports of error.

### Data Analyses

#### Hippocampal subfield volumes

Hemisphere-wise volumetric data (mm^3^) for hippocampal subfields per participant were gathered using FreeSurfer routines (quantifyHippocampalSubfields). Data were analysed in STATA 14 (StataCorp, TX).

#### Statistical modelling

The tightly folded structure of hippocampus leads to high correlation of subfield volumes within and between hemispheres; in part, this may arise via limitations with *in vivo* scan spatial resolution and image contrast, giving reduced spatial accuracy of segmentations. Mixed effects linear regression models of subfield volumes allowed us to fit random effects at the levels of hemisphere and participant, accounting for the intraclass spatial correlation within hemispheres and individuals that arises from these issues. Hence, the random effects modelled subfields as nested within hemispheres, and hemispheres as nested within participants: Y_ijk_ = β_0_ + βn_ijk_… + v_k_ + u_jk_ + e_ijk_; where Y_ijk_ was the volume of hippocampal subfield i, in hemisphere j, within individual k; β_0_ was the model intercept; βn_ijk_… a set of fixed-effect covariate terms; v_k_ the participant-specific intercept; u_jk_ the hemisphere intercept for participant k; and e_ijk_ the subfield-specific residual term. All models included age, age^2^, gender, total grey matter volume, highest level of education (primary, secondary, tertiary), smoker status (never, current-past), handedness, and cardiovascular disease (any history/none of abnormal rhythm, angina, cardiac arrest, or heart attack) as fixed effect covariates. Frailty variables were included with the other covariates in initial models, but were dropped due to lack of improvement in model fit when entered in isolation or together (all *p* > 0.3). Covariates were selected based on previous literature showing impacts of cardiovascular risk^42^, smoking^43^, education^32^, and frailty^44^ on tissue volumes.

In separate models, we tested effects of IR, DR, and AN as predictors of hippocampal subfield volumes. Tasks were modelled separately to avoid multicollinearity. Initial inspection of data suggested non-linear trends between recall performance and subfield volumes. We therefore modelled linear, quadratic, and cubic terms for recall tasks, appraising model fit relative to the next simplest alternative using likelihood ratio tests. Models specified subfield as a fixed term, in addition to subfield being nested in the hemisphere random effect. We hypothesised that subfields including CA1, CA2/3, CA4 and GC-DG would be critical to learning performance, and therefore specified fixed effect linear and non-linear interaction terms between IR/DR and subfield. To explore effects of age comprehensively, we further included fixed effect linear and quadratic interaction terms between age and subfield. Within-model statistical significance (α = 0.05) of all terms was evaluated using Wald tests. All non-linear terms were evaluated with respect to the significance of the related lower order terms; e.g., in the case of quadratic terms, we deemed as significant only those where the corresponding linear term was also significant (since the interpretation of a significant quadratic term in isolation was not meaningful within the present models).

To evaluate the stability of final mixed effect fits for recall tasks, we performed 10-fold cross validation. We used a random sampling procedure to divide the cohort into 10 folds of approximately equal size (6 folds n = 44, 4 folds n = 43); we then iteratively fitted the fully-adjusted mixed models to 90% of the data, holding the remaining 10% for validation. Initial models of 90% of the data were estimated using fixed and random effects; predictions for the held-out 10% sample used the fixed model terms only. Root-mean-square error (RMSE) values were calculated for each of the 10 sets of predictions.

## Results

### Immediate and delayed recall relate to volumes of specific hippocampal subfields

**Figure 1:**
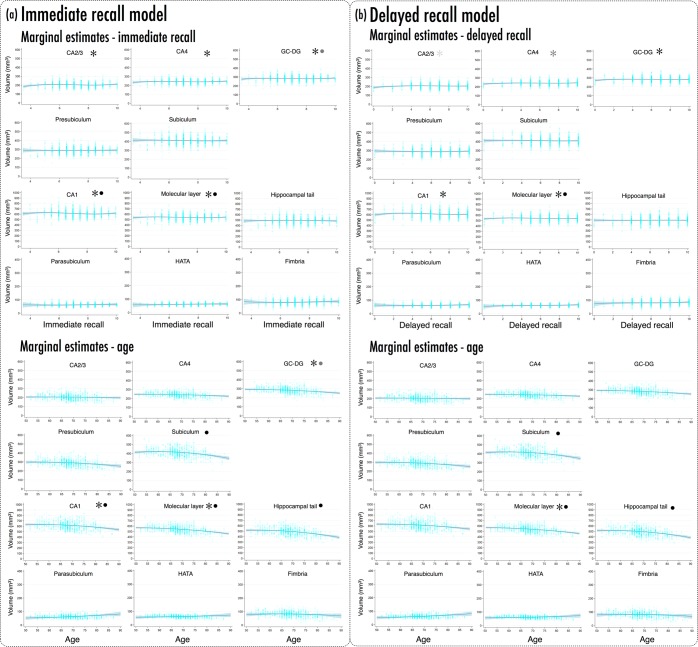
Marginal estimates of hippocampal subfield volumes (mm^3^) from immediate recall (IR) and delayed recall (DR) models, showing effects of IR, DR and age. (**a**) Upper panels present marginal estimates (blue line; light blue band ±95% CI) of subfield volumes as a function of IR score; overlaid cyan scatter presents observed participant-wise data. Lower panels present marginal estimates (±95% CI) of subfield volumes as a function of age (years), with participant-wise scatters. (**b**) Marginal estimates for DR model; all specifications as per (**a**). *IR/DR cubic term significant, *p* < 0.05; • age quadratic term significant, *p* < 0.05; grey shading denotes marginally significant trend - see Tables 2 and 3, and Results. Note differences in y-axis ranges across panel rows in (**a**,**b**); adjusted to accommodate differences in subfield volumes (see also Supplementary Fig. 2). All marginal estimates calculated from fully-adjusted models, holding all covariates at their means.

Using mixed effects linear regression, we analysed hippocampal subfield volumes with respect to recall performance. Figure 1 and Table 2 presents marginal estimates of hippocampal subfield volumes from best-fitting immediate recall (IR) and delayed recall (DR) models, across IR and DR score ranges, and across age bands (note that recall and age estimates incorporate their respective linear and non-linear terms). Table 3 summarises coefficients for the non-linear recall x subfield and age x subfield interaction terms, from the same models (all models fitted main effects for each of the recall and age terms, in addition to their interactions with subfield; see Supplementary Tables S1 and S2 for full IR and DR output, respectively).

**Table 2:** Marginal estimates (95% CI) of hippocampal subfield volumes across immediate recall (IR) and delayed recall (DR) scores, with age effects.

IR model						DR model						
IR score:	4	5.5	7	8.5	10	DR score:	0	2	4	6	8	10
Subfield						Subfield						
**CA2/3**	**195.49 (185.20, 205.78)**	**207.16 (203.13, 211.18)**	**204.06 (201.50, 206.63)**	**200.73 (197.38, 204.08)**	**211.66 (202.54, 220.77)**	*CA2/3*	*188.04 (174.43, 201.65)*	*200.8 (195.14, 206.47)*	*205.34 (201.37, 209.31)*	*205.04 (202.38, 207.71)*	*203.29 (200.13, 206.45)*	*203.46 (197.93, 208.98)*
**CA4**	**238.67 (229.76, 247.57)**	**243.82 (240.32, 247.32)**	**241.12 (238.91, 243.33)**	**239.74 (236.83, 242.65)**	**248.87 (240.98, 256.75)**	*CA4*	*230.99 (219.19, 242.78)*	*240.38 (235.45, 245.32)*	*242.66 (239.21, 246.11)*	*241.53 (239.23, 243.83)*	*240.74 (238.00, 243.47)*	*243.99 (239.20, 248.79)*
**GC-DG**	**280.82 (271.63, 290.02)**	**285.54 (281.93, 289.15)**	**281.68 (279.39, 283.96)**	**279.33 (276.33, 282.33)**	**288.60 (280.46, 296.75)**	**GC-DG**	**271.76 (259.59, 283.94)**	**283.00 (277.91, 288.08)**	**284.94 (281.39, 288.50)**	**282.40 (280.02, 284.77)**	**280.16 (277.33, 282.98)**	**283.02 (278.07, 287.96)**
Presubic.	288.56 (273.70, 303.43)	287.26 (281.50, 293.03)	288.11 (284.39, 291.82)	289.78 (284.97, 294.59)	290.96 (277.80, 304.13)	Presubic.	295.39 (275.81, 314.98)	291.75 (283.66, 299.85)	288.18 (282.48, 293.87)	286.29 (282.44, 290.15)	287.73 (283.18, 292.28)	294.11 (286.15, 302.07)
Subiculum	414.46 (399.76, 429.17)	413.52 (407.82, 419.22)	409.18 (405.51, 412.86)	406.40 (401.64, 411.16)	410.14 (397.11, 423.16)	Subiculum	408.75 (389.36, 428.15)	413.08 (405.06, 421.10)	412.39 (406.75, 418.04)	409.54 (405.72, 413.35)	407.34 (402.83, 411.84)	408.62 (400.74, 416.51)
**CA1**	**619.49 (599.14, 639.84)**	**627.4 (619.55, 635.26)**	**613.44 (608.35, 618.53)**	**602.52 (595.96, 609.09)**	**619.56 (601.54, 637.58)**	**CA1**	**597.09 (570.33, 623.86)**	**624.97 (613.94, 636.00)**	**626.67 (618.90, 634.45)**	**615.56 (610.28, 620.84)**	**605.00 (598.78, 611.22)**	**608.34 (597.46, 619.22)**
**Mol. layer**	**542.20 (527.64, 556.75)**	**548.28 (542.64, 553.93)**	**539.71 (536.07, 543.34)**	**533.16 (528.45, 537.87)**	**545.34 (532.45, 558.23)**	**Mol. layer**	**530.88 (511.71, 550.06)**	**547.18 (539.25, 555.11)**	**547.65 (542.07, 553.23)**	**540.66 (536.89, 544.44)**	**534.60 (530.15, 539.06)**	**537.84 (530.05, 545.64)**
Hipp. tail	486.84 (466.04, 507.63)	493.39 (485.36, 501.42)	493.91 (488.71, 499.12)	490.03 (483.32, 496.74)	483.37 (464.95, 501.78)	Hipp. tail	493.85 (466.44, 521.25)	489.65 (478.36, 500.94)	489.94 (481.98, 497.90)	492.04 (486.63, 497.44)	493.25 (486.88, 499.62)	490.89 (479.75, 502.03)
Parasubic.	61.49 (49.21, 73.77)	60.30 (55.52, 65.08)	61.31 (58.25, 64.38)	63.61 (59.63, 67.60)	66.27 (55.40, 77.14)	Parasubic.	63.61 (47.39, 79.83)	60.82 (54.10, 67.54)	59.93 (55.20, 64.65)	60.70 (57.51, 63.88)	62.90 (59.14, 66.67)	66.31 (59.72, 72.90)
HATA	57.72 (46.12, 69.32)	59.32 (54.80, 63.84)	61.47 (58.58, 64.36)	63.30 (59.53, 67.06)	63.90 (53.63, 74.18)	HATA	53.68 (38.31, 69.04)	58.51 (52.14, 64.89)	60.34 (55.87, 64.82)	60.96 (57.95, 63.97)	62.16 (58.59, 65.72)	65.73 (59.49, 71.97)
Fimbria	81.84 (68.25, 95.43)	78.32 (73.04, 83.59)	79.52 (76.13, 82.92)	83.13 (78.73, 87.53)	86.80 (74.77, 98.83)	Fimbria	73.35 (55.38, 91.33)	78.69 (71.25, 86.13)	80.03 (74.80, 85.26)	79.93 (76.39, 83.46)	80.95 (76.77, 85.13)	85.66 (78.35, 92.97)
**Age:**	**50**	**60**	**70**	**80**		**Age:**	**50**	**60**	**70**	**80**		
**Subfield**						**Subfield**						
CA2/3	205.09 (194.55, 215.64)	205.31 (201.88, 208.73)	203.91 (201.42, 206.39)	200.89 (196.27, 205.52)		CA2/3	205.13 (194.55, 215.71)	205.07 (201.63, 208.50)	203.84 (201.35, 206.33)	201.45 (196.83, 206.07)		
CA4	246.75 (237.61, 255.89)	245.70 (242.71, 248.69)	241.62 (239.48, 243.77)	234.52 (230.47, 238.58)		CA4	246.94 (237.76, 256.12)	245.47 (242.47, 248.47)	241.53 (239.38, 243.68)	235.11 (231.06, 239.17)		
**GC-DG**	**293.64 (284.21, 303.08)**	**290.25 (287.17, 293.32)**	**281.93 (279.71, 284.15)**	**268.69 (264.52, 272.86)**		GC-DG	294.22 (284.75, 303.69)	290.09 (287.00, 293.18)	281.78 (279.56, 284.01)	269.29 (265.12, 273.46)		
Presubic.	298.04 (282.86, 313.23)	296.96 (292.09, 301.83)	288.95 (285.35, 292.55)	274.00 (267.46, 280.55)		Presubic.	298.35 (283.14, 313.56)	297.20 (292.32, 302.07)	288.95 (285.34, 292.55)	273.61 (267.09, 280.13)		
**Subiculum**	**413.04 (398.02, 428.07)**	**420.32 (415.5, 425.14)**	**411.42 (407.85, 414.98)**	**386.33 (379.85, 392.8)**		**Subiculum**	**413.46 (398.39, 428.52)**	**420.14 (415.31, 424.97)**	**411.29 (407.72, 414.86)**	**386.92 (380.45, 393.38)**		
**CA1**	**632.95 (612.19, 653.71)**	**630.61 (623.99, 637.24)**	**613.94 (609.00, 618.88)**	**582.92 (574.05, 591.80)**		CA1	634.80 (614.02, 655.58)	630.20 (623.58, 636.82)	613.49 (608.55, 618.43)	584.67 (575.84, 593.51)		
**Mol. layer**	**568.91 (554.04, 583.79)**	**560.86 (556.08, 565.63)**	**539.54 (536.02, 543.07)**	**504.97 (498.56, 511.38)**		**Mol. layer**	**570.11 (555.21, 585.00)**	**560.68 (555.90, 565.45)**	**539.28 (535.75, 542.81)**	**505.91 (499.52, 512.30)**		
**Hipp. tail**	**517.47 (496.26, 538.69)**	**515.56 (508.79, 522.33)**	**492.92 (487.87, 497.97)**	**449.54 (440.47, 458.61)**		**Hipp. tail**	**515.70 (494.43, 536.97)**	**515.02 (508.25, 521.80)**	**493.11 (488.06, 498.17)**	**449.97 (440.93, 459.02)**		
Parasubic.	51.57 (39.01, 64.13)	56.02 (51.97, 60.07)	62.56 (59.59, 65.53)	71.18 (65.73, 76.64)		Parasubic.	51.61 (39.00, 64.21)	56.10 (52.04, 60.16)	62.57 (59.59, 65.55)	71.02 (65.57, 76.47)		
HATA	56.46 (44.59, 68.33)	58.44 (54.6, 62.28)	61.80 (58.99, 64.61)	66.55 (61.37, 71.72)		HATA	56.46 (44.52, 68.40)	58.42 (54.56, 62.27)	61.80 (58.98, 64.61)	66.60 (61.42, 71.77)		
Fimbria	77.78 (63.89, 91.67)	82.05 (77.58, 86.51)	81.75 (78.46, 85.04)	76.88 (70.87, 82.88)		Fimbria	78.50 (64.54, 92.46)	82.17 (77.69, 86.65)	81.64 (78.34, 84.95)	76.93 (70.92, 82.93)		

Marginal estimates adjusted for sex, education, total brain grey matter volume, handedness, smoking, and cardiovascular disease history. Bold text - significant non-linear effects in fully-adjusted model

(*p* < 0.05); italicised text - marginal linear/non-linear coefficients in fully-adjusted model (*p* < 0.08); see Table 3. See Supplementary Tables 1 and 2 for IR and DR full model outputs, respectively.

**Table 3:** Immediate recall (IR; left) and delayed recall (DR; right) coefficients for model interaction terms - non-linear recall and age effects.

IR terms	Lin. (95% CI)	Quad. (95% CI)	Cub. (95% CI)	Age terms	Lin. (95% CI)	Quad. (95% CI)	DR terms	Lin. (95% CI)	Quad. (95% CI)	Cub. (95% CI)	Age terms	Lin. (95% CI)	Quad. (95% CI)
**CA2/3**	**111.89 (47.73 176.05)**	**−16.35 (−25.72–6.99)**	**0.76 (0.32 1.21)**	CA3	1.61 **(**−2.23 5.46)	−0.02 **(**−0.05 0.01)	**CA2/3**	**10.91 (2.46 19.35)**	**−1.72 (−3.42–0.01)**	0.08 **(**−0.03 0.18)	CA3	1.27 **(**−2.58 5.12)	−0.02 **(**−0.04 0.01)
**CA4**	**69.64 (14.84 124.43)**	**−10.47 (−18.47–2.47)**	**0.5 (0.12 0.88)**	CA4	2.26 **(**−1.02 5.55)	−0.03 **(**−0.05 0.0)	**CA4**	**9.01 (1.79 16.23)**	**−1.62 (−3.08–0.17)**	*0.08 (0.0 0.17)*	CA4	1.85 **(**−1.44 5.14)	−0.02 **(**−0.05 0.0)
**GC-DG**	**74.96 (18.19 131.72)**	**−11.39 (−19.68–3.1)**	**0.54 (0.15 0.94)**	*GC-DG*	*3.07 (−0.33 6.47)*	*−0.04 (−0.06–0.01)*	**GC-DG**	**10.65 (3.17 18.12)**	**−2.03 (−3.53–0.52)**	**0.1 (0.01 0.19)**	GC-DG	2.53 **(**−0.88 5.93)	−0.03 **(**−0.06–0.01)
Presubic.	−1.6 **(**−96.09 92.88)	0.3 **(**−13.5 14.09)	−0.02 **(**−0.67 0.64)	Presubic.	4.41 **(**−1.26 10.07)	−0.05 **(**−0.09 0.0)	Presubic.	0.34 **(**−12.09 12.77)	−0.46 **(**−2.97 2.05)	0.04 **(**−0.11 0.19)	Presubic.	4.42 **(**−1.24 10.08)	−0.05 **(**−0.09 0.0)
Subiculum	37.73 **(**−55.7 131.16)	−6.04 **(**−19.68 7.60)	0.29 **(**−0.36 0.94)	**Subiculum**	**10.33 (4.73 15.93)**	**−0.09 (−0.13–0.05)**	Subiculum	5.79 (−6.51 18.1)	−1.25 **(**−3.73 1.24)	0.06 **(**−0.08 0.21)	**Subiculum**	**9.85 (4.24 15.45)**	**−0.09 (−0.13–0.05)**
**CA1**	**169.87 (39.5 300.24)**	**−26.41 (−45.45–7.37)**	**1.28 (0.37 2.18)**	**CA1**	**8.36 (0.54 16.17)**	**−0.08 (−0.14–0.03)**	**CA1**	**24.62 (7.47 41.76)**	**−5.21 (−8.67–1.75)**	**0.28 (0.08 0.49)**	CA1	6.84 **(**−0.97 14.65)	−0.07 **(**−0.13–0.01)
**Mol. layer**	**117.47 (25.02 209.93)**	**−18.11 (−31.61–4.61)**	**0.87 (0.23 1.51)**	**Mol. layer**	**7.19 (1.65 12.73)**	**−0.08 (−0.12–0.04)**	**Mol. layer**	**15.41 (3.25 27.57)**	**−3.29 (−5.75–0.84)**	**0.18 (0.03 0.33)**	**Mol. layer**	**6.28 (0.74 11.82)**	**−0.07 (−0.11–0.03)**
Hipp. tail	33.73 **(**−99.56 167.03)	−3.91 **(**−23.38 15.55)	0.13 **(**−0.8 1.05)	**Hipp. tail**	**11.92 (3.92 19.91)**	**−0.11 (−0.17–0.06)**	Hipp. tail	−1.76 **(**−19.32 15.8)	0.63 **(**−2.91 4.18)	−0.05 **(**−0.26 0.16)	**Hipp. tail**	**12.25 (4.25 20.25)**	**−0.12 (−0.17–0.06)**
HATA	5.52 **(**−67.37 78.42)	−0.4 **(**−11.04 10.24)	0.0 **(**−0.5 0.51)	HATA	0.14 **(**−4.23 4.51)	0.0 **(**−0.04 0.03)	HATA	5.38 **(**−4.25 15)	−0.87 **(**−2.81 1.08)	0.04 **(**−0.07 0.16)	HATA	0.05 **(**−4.34 4.43)	0.0 **(**−0.03 0.03)
Fimbria	−13.02 **(**−99.07 73.04)	1.71 **(**−10.86 14.27)	−0.07 **(**−0.67 0.53)	Fimbria	3.64 **(**−1.52 8.8)	−0.03 **(**−0.07 0.0)	Fimbria	6.01 **(**−5.36 17.37)	−1.09 **(**−3.38 1.21)	0.06 **(**−0.08 0.2)	Fimbria	3.31 **(**−1.87 8.49)	−0.03 **(**−0.07 0.01)

Bold text - significant coefficients (*p* < 0.05); italicised text - marginal coefficients (*p* < 0.08). Note differences in coefficient magnitudes for IR and DR interaction terms; subfield main effect reached significance in DR model, but not IR model. See Supplementary Tables 1 and 2 for IR and DR full model outputs, respectively.

We appraised whether inclusion of IR terms and their subfield interactions improved model fit. Iterative addition of IR interaction terms to the fully adjusted model revealed that the cubic IR terms and their interaction with subfield significantly improved the model fit, over quadratic (likelihood ratio [LR] test: *χ*^2^_(11)_ = 555.6, *p* < 0.00001) and linear (LR: *χ*^2^_(22)_ = 565.8, *p* < 0.00001) IR and their subfield interaction terms in the model. Cubic IR terms and their interaction with subfield significantly improved the fit of the fully adjusted model, relative to the fully adjusted model with no IR terms (LR: *χ*^2^_(33)_ = 611.0, *p* < 0.00001; ΔAIC: 547). Similarly, in the fully adjusted DR model, the cubic DR and subfield interaction terms improved the fit significantly, compared to the quadratic (LR: *χ*^2^_(11)_ = 552.0, *p* < 0.00001) and linear only (LR: *χ*^2^_(22)_ = 576.95, *p* < 0.00001) DR and subfield interaction terms; the fully adjusted model with cubic DR terms significantly improved the fit relative to that model omitting all DR terms (LR: *χ*^2^_(33)_ = 613.02, *p* < 0.00001; ΔAIC: 547).

In the fully adjusted IR model, significant subfield x cubic IR interactions emerged for CA1 (*p* < 0.006), CA2/3 (*p* < 0.001), CA4 (*p* < 0.01), GC-DG (*p* < 0.007), and molecular layer (*p* < 0.008) (Fig. 1a, top; Tables 2 & 3, left). In the same model, subfield x quadratic age interactions were significant for subiculum (*p* < 0.0001), CA1 (*p* < 0.005), molecular layer (*p* < 0.0001), and hippocampal tail (*p* < 0.0001), with a marginal trend for GC-DG (lin. *p* < 0.08, quad. *p* < 0.005; see Fig. 1a, bottom; Tables 2 & 3, left). Thus, CA2/3 and CA4 showed significant cubic fits for IR but no significant age effects; in contrast, subiculum and hippocampal tail showed significant quadratic age fits, but no significant effects of IR. CA1, molecular layer, and to a degree, GC-DG, manifested effects of IR and age.

A similar pattern emerged for the DR model. Subfield x cubic DR interactions were significant for CA1 (*p* < 0.007), GC-DG (*p* < 0.023), and molecular layer (*p* < 0.016), with a marginally significant cubic term for CA4 (*p* < 0.065); the cubic term did not reach significance for CA2/3 (*p* > 0.1), but the quadratic term was significant (*p* < 0.029) (Fig. 1b, top; Tables 2 & 3, right). Further, subfield x quadratic age interactions were significant for subiculum (*p* < 0.0001), molecular layer (*p* < 0.001), hippocampal tail (*p* < 0.0001), with a weak trend for CA1 (lin. *p* < 0.086, quad. *p* < 0.02; Fig. 1b, bottom; Tables 2 & 3, right). Like the IR model, CA2/3 and CA4 showed significant non-linear effects of DR but no significant age effects, whereas subiculum and hippocampal tail showed significant quadratic age effects but no significant DR effects. Both cubic DR and quadratic age effects manifested at molecular layer, while cubic DR effects were significant at CA1 but age effects were not.

### Verbal fluency shows no robust relationship with hippocampal subfield volumes

We used mixed effects regression to analyse subfield volume with respect to animal naming (AN). As for the IR and DR models, we fitted the interaction between AN and subfield to the fully adjusted model. The subfield x AN linear interaction reached significance for presubiculum only (β = 0.56, *p* < 0.011, 95% CI: 0.13–0.99; other regions all *p* > 0.18). Evaluating the AN model against the fully adjusted model with no AN interaction terms showed no significant improvement in model fit with the addition of the AN terms (LR: LR: *χ*^2^_(11)_ = −489.0, *p* > 0.9; ΔAIC: −511). Quadratic age effects in the model including AN terms recapitulated the IR model, with age effects at presubiculum and fimbria also reaching significance; however, omitting the AN terms yielded age effects consistent with the IR model (see Supplementary Table S3).

### Consistency of IR and DR model predictions

To appraise the stability of the IR and DR models, we employed 10-fold cross validation, using a ‘leave one out’ procedure to initially fit each model whilst the remaining fold was held for validation. We used the fixed effects from the fully-adjusted IR and DR models with cubic recall terms fitted for training and prediction. Table 4 presents the RMSE values across folds for IR and DR predictions. Supporting the pattern of results found for the fully adjusted IR and DR models of the entire sample, cross-validated estimates of prediction error showed good consistency within and across models (IR RMSE range & SD: 36.33–42.13, 2.08; DR RMSE range & SD: 36.38–40.95, 1.51).

**Table 4:** RMSE values for model predictions from 10-fold internal cross-validation of IR and DR models.

Model	Fold 1	Fold 2	Fold 3	Fold 4	Fold 5	Fold 6	Fold 7	Fold 8	Fold 9	Fold 10
IR	38.37	38.25	40.86	41.99	36.60	42.13	38.89	36.33	37.51	38.18
DR	37.57	37.60	39.62	40.95	36.38	40.67	38.18	37.56	37.63	39.45

## Discussion

Here, we examined relationships between volumes of hippocampal subfields and performance in two domains of memory, in healthy ageing. We found that immediate recall (IR) performance during verbal list learning was associated non-linearly with volumes of CA1, CA2/3, CA4, GC-DG, and molecular layer, with cubic terms providing the best fits per subfield. Similarly, subsequent performance on list delayed recall (DR) was associated non-linearly with volumes of these subfields; again, cubic terms tended to afford the best fits (cf. CA2/3). In parallel, we observed age-related decline in the volumes of subiculum, molecular layer, and hippocampal tail in both models, with further declines noted for CA1 and GC-DG in the IR model; age-related declines were best fit by quadratic terms. Finally, we found that semantic fluency was not robustly associated with volumes of hippocampal subfields.

As predicted, our results revealed roles for CA1-CA4 and GC-DG, in addition to molecular layer, in the encoding and subsequent retrieval of novel verbal word lists. Several recent studies have provided piecemeal evidence of associations between subfield volumes and verbal episodic memory, implicating subiculum in immediate verbal recall^45^, and CA1^46^, subiculum^46,47^, and presubiculum^45^ in delayed verbal recall. Our current IR findings implicate each of the CA subfields and GC-DG, supporting their previously demonstrated roles in encoding of novel stimuli/environments^12,34^, and in verbal and visuo-spatial episodic memory^17,48^. Our DR results agree in part with the IR findings; although cubic fit robustness was reduced for CA2/3 and CA4, overall trends showed that DR performance fluctuated non-linearly with CA, GC-DG and molecular layer subfield volume (further to^17^). Augmenting these results, our observation that free recall of familiar semantic categories showed little relationship with subfield volumes agrees with accounts of semantic memory as dissociable to non-hippocampal medial temporal regions, and temporal pole^21,22^.

A major aim of our present analyses was to explore age-related differences in subfield volumes in tandem with memory performance. That age terms in both the IR and DR models revealed non-linear decline in the volumes of subiculum, molecular layer, and hippocampal tail, differs from existing *in vivo*^23^ and *ex vivo*^24^ results, which have shown age-related decline in CA1^23,24^ and dentate gyrus/CA4 volumes^23^. Although we observed some evidence of age-related decline in the IR model for CA1, we note that the GC-DG trend was weaker, and neither CA1 nor GC-DG showed robust decline with age in the DR model. Differences in the segmentation procedures (here, automated; cf.^23^, manual) and our larger sample size likely account for the divergent findings. A notable feature of our present results was the lack of age-related decline for CA2/3 or CA4, whereas volumes of both regions fluctuated with IR and DR performance. Animal models have shown critical roles for dentate gyrus and CA3 in pattern separation and pattern completion respectively, whereby many sources of cortical information are decorrelated in support of discretised memory representations (separation), and where various traces may be combined to allow recall based on multiple representations (completion)^12,13,34,49^. One implication of our findings may be that pattern completion processes focal to CA2/3 and perhaps CA4 are less susceptible to age-related atrophy in health, whereas regions including subiculum are more prone to manifesting grey matter loss^50^. Current clinical evidence suggests CA1-CA4 volumes and related recall performance appear to be most heavily impacted in the progression of MCI and Alzheimer’s disease^51,52^.

Our findings hold broader implications for memory performance and subfield atrophy in healthy ageing. The complexity of the trends observed in our data suggests that prediction of those at risk of eventual memory impairment requires a multiple-subfield view of hippocampus^26^. In particular, the cubic trends noted in our data suggest that poorest performers in IR and DR are likely to manifest subtle tissue loss in subfields including CA2/3, CA4 and GC-DG, compared to average performers. Moreover, it is notable that subfields including CA1 showed fluctuation in volume across IR and DR scores, yet at the upper and lower tails of the IR performance range, CA1 volumes were similar (see Table 2, left). Taken together, our results suggest that detecting those most at risk of subtle memory impairment may require memory assessment at multiple time points, and detailed assessment of CA2/3, CA4 & GC-DG anatomy, against well-characterised normative data for a range of ages. A limitation of the existing literature has been the relatively small sample sizes employed (typically N < 150), which may mask the complexity of performance-anatomy relationships; here, we were able to characterise these profiles in a large sample with broad ranges of both memory performance and age.

Avenues for future research may include the potential to combine detailed assays of hippocampal subfield volumes with advanced machine learning techniques, as a means of predicting cognitive performance based on subfield volumes. Recent machine learning approaches have tracked the progression of MCI towards Alzheimer’s disease (e.g., by training support vector machines to discriminate between Alzheimer’s patients and healthy controls, and then applying the trained model to MCI patient data^53^). However, such approaches are restricted to classification of categorical disease outcomes. More recent approaches have involved predicting continuous data (e.g., age^54,55^, pain ratings^56^) from MRI scans using machine learning methods (e.g., elastic net or Gaussian process regression). Advancing such techniques, recent approaches have trained artificial neural networks to predict cognitive performance based on hippocampal subfield volumes and cortical thickness data^57^. In future studies, such models could be used to generate predictions for an individual’s expected longitudinal cognitive performance; an observed discrepancy between the model prediction and an individual’s subsequent true performance could serve as a clinical indicator for MCI risk. Moreover, the potential to construct such models using a range of additional physiological measurements as training set features (e.g., serum markers for inflammatory cytokines, blood pressure, objective gait assays) could enhance prediction accuracy, by allowing for broader characterisation of both neural and physiological phenotypes that may precede MCI onset.

An important consideration for future studies will be scan spatial resolution, which impacts the accuracy of hippocampal subfield measurements; replication of the present results in a cohort with scans of < 0.6 mm isotropic resolution would be beneficial. Indeed, previous investigations have employed higher resolution scans than the present study^27,58^, within semi- and fully-automated hippocampal segmentation routines. A further issue concerns the image contrast employed in the segmentation protocol^26,58,59^. A number of automated pipelines (including that within FreeSurfer v.6.0) enable the specification of T_1w_ and T_2w_ input images, as aids to hippocampal atlas construction^59^ or subject-level hippocampal segmentation^26^. The present imaging protocol did not include a T_2w_ acquisition that was suitable for combined use with the T_1w_ image (owing to in-plane resolution differences); hence, only the T_1w_ image could be used as input to the FreeSurfer segmentation procedure. This holds implications for the accuracy of segmentation of some subfields. As outlined in^26^, use of T_1w_ images alone in the FreeSurfer parcellation scheme can lead to under segmentation of the molecular layer, an issue that is largely resolved when both T_1w_ and T_2w_ images are used. Thus, future MRI investigations with the TILDA cohort would benefit from integration of high-resolution T_1w_ and T_2w_ scans in order to achieve the most optimal estimates of tissue volumes within the hippocampal subfields.

In sum, our results reveal that specific subfields of the hippocampus manifest non-linear associations with verbal memory encoding and retrieval performance in non-demented older adults. These effects are partly dissociable from age-related atrophy, and from naming of well-consolidated semantic categories. Our results may enable us to generate predictions for those at greatest risk of incident memory impairment in future TILDA waves.

## Supplementary information

Supplementary Materials

## Data Availability

The datasets generated during and/or analysed during the current study are not publicly available due to data protection regulations, but are accessible at TILDA on reasonable request.

## References

[1] Scoville WB, Milner B (1957). Loss of recent memory after bilateral hippocampal lesions. J. Neurol Neurosurg Psychiat..

[2] Squire LR, Zola-Morgan S (1991). The medial temporal lobe memory system. Science..

[3] Squire LR (1992). Memory and the hippocampus: a synthesis from findings with rats, monkeys, and humans. Psych. Rev..

[4] Gordon BA (2016). Longitudinal β-amyloid deposition and hippocampal volume in preclinical alzheimer disease and suspected non–alzheimer disease pathophysiology. JAMA Neurol..

[5] Reilly JF (2003). Amyloid deposition in the hippocampus and entorhinal cortex: quantitative analysis of a transgenic mouse model. Proc. Natl. Acad. Sci. USA.

[6] Schöll M (2016). PET imaging of tau deposition in the aging human brain. Neuron..

[7] Wolk DA, Das SR, Mueller SG, Weiner MW, Yushkevich PA (2017). Medial temporal lobe subfieldal morphometry using high resolution MRI in Alzheimer’s disease. Neurobiol. Aging..

[8] Amunts K (2005). Cytoarchitectonic mapping of the human amygdala, hippocampal region and entorhinal cortex: intersubject variability and probability maps. Anat. Embryol..

[9] Adler DH (2014). Histology-derived volumetric annotation of the human hippocampal subfields in postmortem MRI. NeuroImage..

[10] Lee I, Yoganarasimha D, Rao G, Knierim JJ (2004). Comparison of population coherence of place cells in hippocampal subfields CA1 and CA3. Nature..

[11] Lee I, Rao G, Knierim JJ (2004). A double dissociation between hippocampal subfields: differential time course of CA3 and CA1 place cells for processing changed environments. Neuron..

[12] Leutgeb JK, Leutgeb S, Moser MB, Moser EI (2007). Pattern separation in the dentate gyrus and CA3 of the hippocampus. Science..

[13] Bakker A, Kirwan CB, Miller M, Stark CE (2008). Pattern separation in the human hippocampal CA3 and dentate gyrus. Science..

[14] Ranganath C, D’Esposito M (2001). Medial temporal lobe activity associated with active maintenance of novel information. Neuron..

[15] Gabrieli JD, Brewer JB, Desmond JE, Glover GH (1997). Separate neural bases of two fundamental memory processes in the human medial temporal lobe. Science..

[16] Peter J (2018). Real-world navigation in amnestic mild cognitive impairment: The relation to visuospatial memory and volume of hippocampal subfields. Neuropsychologia..

[17] Travis SG (2014). High field structural MRI reveals specific episodic memory correlates in the subfields of the hippocampus. Neuropsychologia..

[18] Boucher O, Dagenais E, Bouthillier A, Nguyen DK, Rouleau I (2015). Different effects of anterior temporal lobectomy and selective amygdalohippocampectomy on verbal memory performance of patients with epilepsy. Epilepsy Behav..

[19] Sass KJ (1992). Specificity in the correlation of verbal memory and hippocampal neuron loss: dissociation of memory, language, and verbal intellectual ability. J. Clin. Exp. Neuropsych..

[20] Davis MH, Di Betta AM, MacDonald MJ, Gaskell MG (2009). Learning and consolidation of novel spoken words. J. Cognitive Neurosci..

[21] Mummery CJ (2000). A voxel‐based morphometry study of semantic dementia: relationship between temporal lobe atrophy and semantic memory. Ann. Neurol..

[22] Chan D (2001). Patterns of temporal lobe atrophy in semantic dementia and Alzheimer’s disease. Ann. Neurol..

[23] Wisse LE (2014). Hippocampal subfield volumes at 7T in early Alzheimer’s disease and normal aging. Neurobiol. Aging..

[24] Šimić G, Kostović I, Winblad B, Bogdanović N (1997). Volume and number of neurons of the human hippocampal formation in normal aging and Alzheimer’s disease. J. Comp. Neurol..

[25] Krogsrud SK (2014). Development of hippocampal subfield volumes from 4 to 22 years. Hum. Brain. Mapp..

[26] Iglesias JE (2015). A computational atlas of the hippocampal formation using *ex vivo*, ultra-high resolution MRI: application to adaptive segmentation of *in vivo* MRI. NeuroImage..

[27] Yushkevich PA (2010). Nearly automatic segmentation of hippocampal subfields in *in vivo* focal T_2_-weighted MRI. NeuroImage..

[28] Pluta J, Yushkevich P, Das S, Wolk D (2012). *In vivo* analysis of hippocampal subfield atrophy in mild cognitive impairment via semi-automatic segmentation of T2-weighted MRI. J. Alzheimers. Dis..

[29] Whelan CD (2016). Heritability and reliability of automatically segmented human hippocampal formation subfields. NeuroImage..

[30] Ho NF (2017). Progression from selective to general involvement of hippocampal subfields in schizophrenia. Mol. Psychiatr..

[31] Cao B (2017). Hippocampal subfield volumes in mood disorders. Mol. Psychiatr..

[32] Kang DW, Lim HK, Joo SH, Lee NR, Lee CU (2018). The association between hippocampal subfield volumes and education in cognitively normal older adults and amnestic mild cognitive impairment patients. Neuropsych. Dis. Treat..

[33] Prince M (2013). The global prevalence of dementia: a systematic review and metaanalysis. Alzheimers Dement..

[34] Golomb J (1996). Hippocampal formation size predicts declining memory performance in normal ageing. Neurology..

[35] Lee I, Kesner RP (2004). Encoding versus retrieval of spatial memory: double dissociation between the dentate gyrus and the perforant path inputs into CA3 in the dorsal hippocampus. Hippocampus..

[36] Kenny, R.A. *et al*. The design of the Irish Longitudinal Study on Ageing. TILDA, tilda.tcd.ie/publications/reports/pdf/Report_DesignReport.pdf (2010).

[37] Whelan BJ, Savva GM (2013). Design and methodology of the Irish Longitudinal Study on Ageing. J. Am. Geriatr. Soc..

[38] Shih RA, Lee J, Das L (2011). Harmonization of cross-national studies of ageing to the Health and Retirement Study. RAND Lab Pop..

[39] Dale AM, Fischl B, Sereno MI (1999). Cortical surface-based analysis: I. Segmentation and surface reconstruction. NeuroImage..

[40] Fischl B, Sereno MI, Dale AM (1999). Cortical surface-based analysis: II: inflation, flattening, and a surface-based coordinate system. NeuroImage..

[41] Fischl B, Sereno MI, Tootell RB, Dale AM (1999). High-resolution intersubject averaging and a coordinate system for the cortical surface. Hum. Brain. Mapp..

[42] Debette S (2011). Midlife vascular risk factor exposure accelerates structural brain aging and cognitive decline. Neurology..

[43] Gallinat J (2006). Smoking and structural brain deficits: a volumetric MR investigation. Eur J Neurosci..

[44] Buchman AS, Schneider JA, Leurgans S, Bennett DA (2008). Physical frailty in older persons is associated with Alzheimer’s disease pathology. Neurology..

[45] Ørbo MC, Vangberg TR, Tande PM, Anke A, Aslaksen PM (2018). Memory performance, global cerebral volumes and hippocampal subfield volumes in long-term survivors of out-of-hospital cardiac arrest. Resuscitation..

[46] Zammit AR (2017). Roles of hippocampal subfields in verbal and visual episodic memory. Behav. Brain Res..

[47] Voets NL, Hodgetts CJ, Sen A, Adcock JE, Emir U (2017). Hippocampal MRS and subfield volumetry at 7T detects dysfunction not specific to seizure focus. Sci. Rep..

[48] Tamnes CK (2014). Regional hippocampal volumes and development predict learning and memory. Dev. Neurosci-Basel..

[49] Duzel E, van Praag H, Sendtner M (2016). Can physical exercise in old age improve memory and hippocampal function?. Brain..

[50] Kurth F, Cherbuin N, Luders E (2015). Reduced age-related degeneration of the hippocampal subiculum in long-term meditators. Psychiat. Res-Neuroim..

[51] Carlesimo GA (2015). Atrophy of presubiculum and subiculum is the earliest hippocampal anatomical marker of Alzheimer’s disease. Alzheimers Dement..

[52] Kerchner GA (2012). Hippocampal CA1 apical neuropil atrophy and memory performance in Alzheimer’s disease. NeuroImage..

[53] Costafreda SG (2011). Automated hippocampal shape analysis predicts the onset of dementia in mild cognitive impairment. NeuroImage..

[54] Cole JH (2018). Brain age predicts mortality. Molec. Psychiatr..

[55] Franke K, Gaser C (2012). Longitudinal changes in individual BrainAGE in healthy aging, Mild Cognitive Impairment, and Alzheimer’s Disease. GeroPsych..

[56] Lee J (2019). Machine learning–based prediction of clinical pain using multimodal neuroimaging and autonomic metrics. Pain..

[57] Bhagwat Nikhil, Pipitone Jon, Voineskos Aristotle N., Chakravarty M. Mallar (2019). An artificial neural network model for clinical score prediction in Alzheimer disease using structural neuroimaging measures. Journal of Psychiatry and Neuroscience.

[58] Yushkevich PA (2015). Automated volumetry and regional thickness analysis of hippocampal subfields and medial temporal cortical structures in mild cognitive impairment. Hum Brain Mapp..

[59] Wisse LE, Biessels GJ, Geerlings MI (2014). A critical appraisal of the hippocampal subfield segmentation package in FreeSurfer. Frontiers Aging Neurosci..

