# Supplementary material for: Dissociable age and memory relationships with hippocampal subfield volumes in vivo:Data from the Irish Longitudinal Study on Ageing (TILDA)
Source: Sci Rep. 2019 Jul 29;9:10981. doi: 10.1038/s41598-019-46481-5 (PMC6662668; doi:10.1038/s41598-019-46481-5)
Supplement: Supplementary file 1 — Supplementary Materials [file 41598_2019_46481_MOESM1_ESM.pdf]

Dissociable age and memory relationships with hippocampal subfield volumes in vivo: Data from the Irish Longitudinal Study on Ageing (TILDA)

Daniel Carey [1,2 *]

Hugh Nolan [1,2]

Rose Anne Kenny [1,2]

James Meaney [3,4]

Supplementary Materials

1. The Irish Longitudinal Study on Ageing (TILDA), Trinity College Dublin, Dublin 2, Ireland
2. Dept. of Medical Gerontology, Trinity College Dublin, Dublin 2, Ireland
3. Dept. of Clinical Radiology, Trinity College Dublin, Dublin 2, Ireland
4. Centre for Advanced Medical Imaging (CAMI), St. James' Hospital, Dublin 8, Ireland

Supplementary Methods

1. Verbal Episodic Memory and Semantic Fluency Assessment

The CAPI immediate recall (IR) and delayed recall (DR) tasks were each adopted from the Health and Retirement Study (HRS), a large prospective cohort study of retired older adults resident in the United States (note that the HRS cohort was merged with the cohort of the Asset Health Dynamics Among the Oldest Old (AHEAD) study in 1998) [1,2]. The animal naming task was adapted from the English Longitudinal Study on Ageing (ELSA), a prospective cohort study of older adults resident in the United Kingdom [3]. TILDA is harmonised with both HRS and ELSA, and thus many measures used in those studies have been adopted in largely identical form by TILDA [4].

IR and DR tasks. Briefly, the IR and DR tasks comprise four 10-item word lists, consisting of common nouns ranging in length from one to two syllables. The word lists employed in the HRS IR and DR tasks have subsequently been adopted in unedited form by several international prospective cohort studies of ageing, including the English Longitudinal Study on Ageing (ELSA) and the Irish Longitudinal Study on Ageing (TILDA). Validation of the IR and DR wordlist items was reported by [1], including the factor structure of the IR and DR tasks and normative data for task performance, across multiple years of study data from HRS and the AHEAD study (which included the same IR and DR tasks and wordlists as HRS). With respect to factor structure, IR and DR tasks from AHEAD years 1993 and 1995, and HRS year 1996, were found to load highly on a single factor (factor score ranges, AHEAD 1993 & 1995: IR,

0.94-0.95; DR, 0.76-0.79; factor scores, HRS 1996: IR, 0.94; DR, 0.76; see [1], their Table 12). Further, in exploring relationships across HRS cognitive tasks (data from Waves 8-10 of HRS/AHEAD, 2006-2010), [2] reported the correlation between IR and DR as 0.76, $p<0.01$ (their Table 3). Indeed, this agrees closely with the correlation observed in the present TILDA Wave 3 MRI cohort between the IR and DR tasks ($\rho=0.7$, $p<0.0001$).

Normative IR and DR data (all scale ranges: 0-10) presented by [1] for HRS years 1992 and 1994 revealed the following means (±SDs) for total scores (their Table 7): 1992 – IR, 7.61 (2.65) [N=9090]; DR, 5.54 (2.83) [N=9020]. 1994 – IR, 8.09 (3.07) [N=7968]; DR, 6.22 (3.25) [N=7832]. Similarly, normative data presented by [3] for IR and DR in Wave 4 of ELSA (2008-2009) showed means (±SDs) as follows (their Table 1): IR, 5.7 (1.7); DR, 4.3 (2.0); [both N=5197]. The means and standard deviations reported above are in accordance with the medians and interquartile ranges found for the TILDA MRI sample in the current report (see Table 1; median IR (IQR): 7 (6.5, 8.5); median DR (IQR): 7 (5, 8) [N=436]).

As demonstrated above, across different years from the HRS/AHEAD prospective cohort studies of older adults, the IR and DR tasks revealed consistent factor structures, indicative of strong construct validity and good reliability. In utilising the same task wordlists and closely aligned procedure, correlations between the IR and DR tasks were found to be of similar magnitude in TILDA as in ELSA. Moreover, normative data for performance on IR and DR suggested similar means/medians and ranges of performance

across the three studies, notably with TILDA median IR scores falling between the means of ELSA and HRS.

Animal naming task. The animal naming task entails self-generation of common semantic categories by participants, and is routinely used in the evaluation of clinical populations, including patients with Alzheimer's-type dementia [5], and in patients with neuropsychological conditions [6]. Adopting the procedure of ELSA, participants in TILDA were asked to name as many animals as possible within 60 seconds (discounting proper nouns and repetitions).

In a systematic review of the extant literature on verbal semantic fluency evaluation in large cohort studies, [6] reported the mean (±SD) scores from animal naming tasks conducted in 12 studies carried out between the years 1999 and 2015. The mean of mean scores from the 12 studies was reported as 19.83 (5.76) animals named correctly [6] (their Table 1) (we note that the 12 studies included one on the TILDA cohort; see [7]). Furthermore, examining verbal semantic fluency in the ELSA cohort (Wave 4, 2008-2009), [3] found mean (±SD) of animal naming scores was 20.3 (6.5) [N=5197], comparable to means (±SDs) reported previously by [8] for Wave 1 (20.8±7.0) and Wave 2 (19.2±6.1) of TILDA [N=3417]. Indeed, the current MRI sub-cohort of TILDA (randomly sampled from the main cohort) had a median animal naming score of 19 (IQR: 15.5,23), in line with the Wave 1 and Wave 2 observations for the sample of the main TILDA cohort analysed by [8], and the means reported by [3].

Hence, given the close consistency of central tendency measurements for animal naming from a sample of large cohort studies [6], ELSA [3], and previous TILDA Waves [8], the results from the present report concur closely with expected score distributions.

Test-retest reliability: TILDA MRI cohort. Finally, appraising the test-retest reliability of the IR, DR and AN tasks within the TILDA MRI cohort, we ran Spearman correlations across these tasks for participants within the MRI cohort who had provided data for all three tasks at Waves 1-3 of TILDA. In order to maximise the test-retest N(=513), we included participants in the correlations who were excluded from the mixed effects analyses in the present report based on MRI QC criteria (e.g., lesions, motion artifacts, poor reconstructions). Since the IR task in each wave allowed participants two attempts at recalling the word list, we entered each attempt of the IR task per wave into the correlational analyses, in order to appraise consistency of measurement within the same session (attempts referred to as IR1 and IR2).

Supplementary table 4 presents the test-retest correlations for the IR, DR and AN tasks, across Waves 1 to 3. As can be seen for the IR task, the completion attempts within the same session (i.e., IR1 & IR2) were highly correlated; this was true across each of the three waves (all rho>0.6). Similarly, within each wave, the DR task showed moderate to high correlations with both IR1 and IR2 (rho range: 0.54-0.72). Considering between-wave correlations, IR1 and IR2 were moderately correlated across adjacent waves. For instance, correlations between IR1 and IR2 in Waves 1

and Wave 2 ranged from 0.38 to 0.47; similarly, correlations between IR1 and IR2 in Waves 2 and 3 ranged from 0.41 to 0.44. Inspecting DR correlations across waves revealed a similar pattern of moderate correlations between adjacent waves: Wave 1 DR vs. Wave 2 DR, rho=0.53; Wave 2 DR vs. Wave 3 DR, rho=0.53. For IR1 and IR2, correlations across the non-adjacent waves (1 and 3) were reduced (rho range: 0.35-0.39); however, DR correlations remained moderate when considering Wave 1 and Wave 3 (rho=0.51). The AN task was attempted by participants once in each wave; correlations across waves were within the moderate range, and as above, were highest when considering adjacent waves (AN Wave 1 vs. AN Wave 2, rho=0.49; AN Wave 2 vs. AN Wave 3, rho=0.5; AN Wave 1 vs. AN Wave 3, rho=0.44).

In sum, correlational analyses revealed strong test-retest reliability within-wave for assays of IR performance; DR showed similarly high consistency with within-wave IR scores. Across waves, test-retest reliability was strongest for IR performance when considering adjacent waves, but was reduced for non-adjacent waves (likely due to the greater inter-assessment interval); DR performance showed good reliability across adjacent and non-adjacent waves. AN performance across waves suggested good test-retest reliability, with a small increase in correlation between adjacent versus non-adjacent waves. Coupled with the correlations between tasks observed in independent cohorts referenced above (see [2]), our findings point towards largely strong reliability of measurements within the TILDA MRI cohort.

2. Covariate Selection

As presented in Table 1, we measured depressive symptoms using the abbreviated form of the CES-D depression inventory. We found that the range of scores for depressive symptoms was truncated, with the 1st to 3rd quartiles (n=335) of the distribution reflecting scores less than 5. The 4th quartile (n=97) comprised scores of 5 or greater; however, in practice, we found that most of these participants had scores between 5 and 7, below the cutoff of 8 deemed clinically relevant for depression. Hence, the cohort overwhelmingly did not meet criteria for depression. Given the very restricted range of values for CES-D scores and the largely non-depressed nature of the cohort, we did not adjust for CES-D depression scores in statistical models.

References

1. Ofstedal, M.B., Fisher, G.G., Herzog, A.R. Documentation of cognitive functioning measures of the Health and Retirement Study. *HRS Documentation Report.* **DR006,** 1-55 (2005). http://hrsonline.isr.umich.edu/sitedocs/userg/dr-006.pdf

2. Blankson, A.N., McArdle, J.J. A brief report on the factor structure of the cognitive measures in the HRS/AHEAD studies. *J. Aging. Res.* **2014,** 1-5 (2014). doi: 10.1155/2014/798514.

3. Smith, T. O., Neal, S. R., Peryer, G., Sheehan, K. J., Tan, M. P., Myint, P. K. Orientation and verbal fluency in the English Longitudinal Study of Ageing: modifiable risk factors for falls? *Int. Psychogeriatr.* 1-8 (2018). doi: 10.1017/S1041610218002065.

4. Shih, R.A., Lee, J. & Das, L. Harmonization of cross-national studies of ageing to the Health and Retirement Study. *RAND Lab Pop.* **WR-861/7,** 1-101 (2011).

5. Ober, B. A., Dronkers, N. F., Koss, E., Delis, D. C., Friedland, R. P. Retrieval from semantic memory in Alzheimer-type dementia. *J. Clin. Exp. Neuropsyc.* **8(1),** 75-92 (1986).

6. Woods, D. L., Wyma, J. M., Herron, T. J., Yund, E. W. Computerized analysis of verbal fluency: Normative data and the effects of repeated testing, simulated malingering, and traumatic brain injury. *PloS one.* **11(12),** e0166439 (2016).

7. Feeney, J., Finucane, C., Savva, G. M., Cronin, H., Beatty, S., Nolan, J. M., & Kenny, R. A. Low macular pigment optical density is associated with lower cognitive performance in a large, population-based sample of older adults. *Neurobiol aging*. **34(11),** 2449-2456 (2013).

8. Feeney, J., O'Leary, N., & Kenny, R. A. Impaired orthostatic blood pressure recovery and cognitive performance at two-year follow up in older adults: The Irish Longitudinal Study on Ageing. *Clin. Auton. Res.* **26(2),** 127-133 (2016).

9. Rosetti, H.C., Lacritz, L.H., Cullum, C.M., Weiner, M.F. Normative data for the Montreal Cognitive Assessment (MoCA) in a population-based sample. *Neurology*. **13,** 1272-1275 (2011).

Supplementary table 1: Immediate recall (IR) fully adjusted model output

Term	Estimate	Std. Err.	z	*p*	95% CI Lower	95% CI Upper
Subregion (ref: Parasubic.)						
Presubic.	142.1935	142.7524	1.0	0.319	-137.5961	421.9831
Subiculum	6.097465	141.1687	0.04	0.966	-270.588	282.783
CA1	37.05575	196.9797	0.19	0.851	-349.0173	423.1288
CA3	-123.2123	96.93764	-1.27	0.204	-313.2066	66.78199
CA4	0.7526116	82.79014	0.01	0.993	-161.5131	163.0183
Mol. layer	116.8182	139.6909	0.84	0.403	-156.9708	390.6073
HATA	-12.44896	110.136	-0.11	0.91	-228.3116	203.4137
Fimbria	-41.95398	130.0188	-0.32	0.747	-296.7861	212.8782
GC-DG	23.08426	85.76469	0.27	0.788	-145.0115	191.18
Hipp. tail	70.64821	201.3977	0.35	0.726	-324.0841	465.3805
IR	-9.51344	40.32164	-0.24	0.813	-88.54239	69.51551
IR^2	1.246418	5.890191	0.21	0.832	-10.29815	12.79098
IR^3	-0.0457722	0.2797392	-0.16	0.87	-0.5940511	0.5025066
Subregion x IR						
Presubic.	-1.604872	48.20532	-0.03	0.973	-96.08557	92.87583
Subiculum	37.73244	47.67051	0.79	0.429	-55.70005	131.1649
CA1	169.8731	66.51704	2.55	0.011	39.50213	300.2441
CA3	111.8887	32.73437	3.42	0.001	47.73048	176.0469
CA4	69.63506	27.95697	2.49	0.013	14.8404	124.4297
Mol. layer	117.4734	47.17148	2.49	0.013	25.01901	209.9278
HATA	5.522907	37.19127	0.15	0.882	-67.37064	78.41645
Fimbria	-13.01674	43.90537	-0.3	0.767	-99.06969	73.03621
GC-DG	74.95629	28.96143	2.59	0.01	18.19292	131.7197
Hipp. tail	33.73013	68.00896	0.5	0.62	-99.56498	167.0252
Subregion x IR^2						
Presubic.	0.2985861	7.03892	0.04	0.966	-13.49744	14.09462
Subiculum	-6.041867	6.960828	-0.87	0.385	-19.68484	7.601104
CA1	-26.41109	9.712789	-2.72	0.007	-45.4478	-7.374369
CA3	-16.35376	4.779858	-3.42	0.001	-25.72211	-6.985406

Term	Estimate	Std. Err.	z	*p*	95% CI Lower	95% CI Upper
CA4	-10.46946	4.082265	-2.56	0.01	-18.47055	-2.468366
Mol. layer	-18.10557	6.887959	-2.63	0.009	-31.60572	-4.605423
HATA	-0.399259	5.430653	-0.07	0.941	-11.04314	10.24462
Fimbria	1.705601	6.411043	0.27	0.79	-10.85981	14.27102
GC-DG	-11.38945	4.228936	-2.69	0.007	-19.67801	-3.100891
Hipp. tail	-3.912929	9.930638	-0.39	0.694	-23.37662	15.55076
Subregion x IR^3						
Presubic.	-0.0190465	0.3342258	-0.06	0.955	-0.674117	0.6360239
Subiculum	0.2906227	0.3305177	0.88	0.379	-0.3571801	0.9384255
CA1	1.27627	0.4611878	2.77	0.006	0.3723587	2.180182
CA3	0.7625773	0.2269598	3.36	0.001	0.3177444	1.20741
CA4	0.4989833	0.1938363	2.57	0.01	0.1190712	0.8788954
Mol. layer	0.8700831	0.3270577	2.66	0.008	0.2290618	1.511104
HATA	0.001934	0.2578611	0.01	0.994	-0.5034645	0.5073326
Fimbria	-0.0694307	0.3044126	-0.23	0.82	-0.6660684	0.5272069
GC-DG	0.5448519	0.2008006	2.71	0.007	0.15129	0.9384138
Hipp. tail	0.1261307	0.4715318	0.27	0.789	-0.7980548	1.050316
Age	-0.703256	2.42525	-0.29	0.772	-5.45666	4.050148
Age^2	0.0104371	0.0176027	0.59	0.553	-0.0240635	0.0449377
Subregion x age						
Presubic.	4.408865	2.890255	1.53	0.127	-1.25593	10.07366
Subiculum	10.33173	2.858189	3.61	0.0001	4.729778	15.93367
CA1	8.357675	3.988173	2.1	0.036	0.5409989	16.17435
CA3	1.6128	1.96266	0.82	0.411	-2.233943	5.459543
CA4	2.261464	1.676221	1.35	0.177	-1.023869	5.546796
Mol. layer	7.191145	2.828268	2.54	0.011	1.647841	12.73445
HATA	0.1402486	2.229883	0.06	0.95	-4.230242	4.510739
Fimbria	3.64341	2.632442	1.38	0.166	-1.516081	8.802901
GC-DG	3.07018	1.736445	1.77	0.077	-0.3331909	6.47355
Hipp. tail	11.91552	4.077624	2.92	0.003	3.923521	19.90751

Term	Estimate	Std. Err.	z	*p*	95% CI Lower	95% CI Upper
Subregion x age^2						
Presubic.	-0.0451059	0.0209558	-2.15	0.031	-0.0861785	-0.0040333
Subiculum	-0.0913529	0.0207233	-4.41	0.0001	-0.1319699	-0.050736
CA1	-0.0821439	0.0289163	-2.84	0.005	-0.1388187	-0.025469
CA3	-0.01851	0.0142303	-1.3	0.193	-0.0464008	0.0093808
CA4	-0.0255582	0.0121534	-2.1	0.035	-0.0493785	-0.0017378
Mol. layer	-0.0767396	0.0205064	-3.74	0.0001	-0.1169314	-0.0365479
HATA	-0.0035208	0.0161678	-0.22	0.828	-0.0352091	0.0281675
Fimbria	-0.0332859	0.0190865	-1.74	0.081	-0.0706948	0.004123
GC-DG	-0.0350417	0.0125901	-2.78	0.005	-0.0597178	-0.0103655
Hipp. tail	-0.1141039	0.0295648	-3.86	0.0001	-0.1720499	-0.0561579
Total grey matter vol.	0.0003172	0.0000242	13.12	0.0001	0.0002698	0.0003646
Sex (ref: male)	1.20943	2.268755	0.53	0.594	-3.237249	5.656108
Education (ref: primary/none)						
Secondary	-3.404605	2.434367	-1.4	0.162	-8.175876	1.366666
Third/higher	-4.306848	2.500736	-1.72	0.085	-9.2082	0.5945038
Current/past smoker (ref: never smoked)	2.108114	1.798607	1.17	0.241	-1.417091	5.633318
Handedness (ref: right)	-2.509039	3.196911	-0.78	0.433	-8.77487	3.756791
Cardiac disease (ref: none)	-6.24081	2.507516	-2.49	0.013	-11.15545	-1.326169
Intercept	-102.0963	120.4873	-0.85	0.397	-338.247	134.0545
Random effects parameters						
Participant var	218.7309	24.13201	N/A	N/A	176.1973	271.532
Hemisphere var	233.7628	16.19177	N/A	N/A	204.0874	267.7531
Residuals var						
Presubic.	1232.518	59.50633	N/A	N/A	1121.236	1354.845
Subiculum	1191.584	57.69828	N/A	N/A	1083.698	1310.211
Parasubic.	622.5802	29.92984	N/A	N/A	566.5976	684.0941
CA1	2909.599	139.8057	N/A	N/A	2648.092	3196.931

Term	Estimate	Std. Err.	z	*p*	95% CI Lower	95% CI Upper
CA3	232.8506	11.28506	N/A	N/A	211.7503	256.0535
CA4	1.380382	1.537078	N/A	N/A	0.1556583	12.24127
Mol. layer	1153.8	55.7241	N/A	N/A	1049.593	1268.353
HATA	481.6481	23.22459	N/A	N/A	438.2135	529.3879
Fimbria	916.3263	44.30098	N/A	N/A	833.4849	1007.402
GC-DG	47.02224	2.406808	N/A	N/A	42.53388	51.98423
Hipp. tail	3069.823	147.0879	N/A	N/A	2794.659	3372.081

Supplementary table 2: Delayed recall (DR) fully adjusted model output

Term	Estimate	Std. Err.	z	*p*	95% CI Lower	95% CI Upper
Subregion (ref: Parasubic.)						
Presubic.	144.2423	98.89328	1.46	0.145	-49.58499	338.0695
Subiculum	86.18166	97.90113	0.88	0.379	-105.701	278.0643
CA1	399.7109	136.4101	2.93	0.003	132.3521	667.0698
CA3	111.9922	67.19756	1.67	0.096	-19.71257	243.697
CA4	146.3315	57.40157	2.55	0.011	33.82647	258.8365
Mol. layer	368.6315	96.74156	3.81	0.0001	179.0216	558.2415
HATA	-0.1482124	76.57323	0.0	0.998	-150.229	149.9326
Fimbria	-70.53908	90.41317	-0.78	0.435	-247.7456	106.6675
GC-DG	181.4862	59.44923	3.05	0.002	64.96785	298.0046
Hipp. tail	142.3676	139.7302	1.02	0.308	-131.4986	416.2337
DR	-1.908422	5.330646	-0.36	0.72	-12.3563	8.539453
DR^2	0.266382	1.075259	0.25	0.804	-1.841087	2.373851
DR^3	-0.0048573	0.0641651	-0.08	0.94	-0.1306187	0.120904
Subregion x DR						
Presubic.	0.3440867	6.34215	0.05	0.957	-12.0863	12.77447
Subiculum	5.794226	6.278522	0.92	0.356	-6.511452	18.0999
CA1	24.61621	8.74815	2.81	0.005	7.470148	41.76227
CA3	10.90779	4.309464	2.53	0.011	2.461395	19.35418
CA4	9.01003	3.681234	2.45	0.014	1.794943	16.22512
Mol. layer	15.41191	6.204158	2.48	0.013	3.251989	27.57184
HATA	5.376558	4.910737	1.09	0.274	-4.248309	15.00143
Fimbria	6.005151	5.79831	1.04	0.3	-5.359327	17.36963
GC-DG	10.64524	3.812554	2.79	0.005	3.172767	18.1177
Hipp. tail	-1.760542	8.961072	-0.2	0.844	-19.32392	15.80284
Subregion x DR^2						
Presubic.	-0.4619335	1.28024	-0.36	0.718	-2.971158	2.047291
Subiculum	-1.246305	1.267396	-0.98	0.325	-3.730355	1.237746
CA1	-5.208047	1.76592	-2.95	0.003	-8.669187	-1.746907
CA3	-1.716611	0.8699175	-1.97	0.048	-3.421618	-0.0116037

Term	Estimate	Std. Err.	z	*p*	95% CI Lower	95% CI Upper
CA4	-1.622864	0.7431018	-2.18	0.029	-3.079317	-0.1664114
Mol. layer	-3.292506	1.252385	-2.63	0.009	-5.747135	-0.8378774
HATA	-0.8662525	0.9912918	-0.87	0.382	-2.809149	1.076644
Fimbria	-1.086774	1.170459	-0.93	0.353	-3.380832	1.207284
GC-DG	-2.026433	0.7696102	-2.63	0.008	-3.534842	-0.5180251
Hipp. tail	0.6305962	1.808901	0.35	0.727	-2.914785	4.175977
Subregion x DR^3						
Presubic.	0.0387764	0.0764449	0.51	0.612	-0.1110528	0.1886057
Subiculum	0.0638613	0.075678	0.84	0.399	-0.0844648	0.2121874
CA1	0.2831935	0.1054455	2.69	0.007	0.0765241	0.489863
CA3	0.0753015	0.051944	1.45	0.147	-0.0265069	0.1771098
CA4	0.082498	0.0443716	1.86	0.063	-0.0044688	0.1694648
Mol. layer	0.1793963	0.0747816	2.4	0.016	0.0328271	0.3259656
HATA	0.0422152	0.0591914	0.71	0.476	-0.0737978	0.1582282
Fimbria	0.0582368	0.0698897	0.83	0.405	-0.0787446	0.1952182
GC-DG	0.1047487	0.0459545	2.28	0.023	0.0146795	0.1948178
Hipp. tail	-0.0511063	0.108012	-0.47	0.636	-0.2628059	0.1605933
Age	-0.6375024	2.427431	-0.26	0.793	-5.39518	4.120176
Age^2	0.0098815	0.0176103	0.56	0.575	-0.024634	0.044397
Subregion x age						
Presubic.	4.422115	2.889023	1.53	0.126	-1.240266	10.0845
Subiculum	9.84731	2.860039	3.44	0.001	4.241737	15.45288
CA1	6.83658	3.985022	1.72	0.086	-0.9739185	14.64708
CA3	1.270633	1.963078	0.65	0.517	-2.57693	5.118196
CA4	1.851111	1.676903	1.1	0.27	-1.435559	5.13778
Mol. layer	6.276305	2.826163	2.22	0.026	0.7371265	11.81548
HATA	0.0486649	2.236975	0.02	0.983	-4.335725	4.433055
Fimbria	3.310284	2.641289	1.25	0.21	-1.866547	8.487114
GC-DG	2.527191	1.736722	1.46	0.146	-0.876722	5.931105
Hipp. tail	12.24746	4.082013	3.0	0.003	4.246856	20.24805

Term	Estimate	Std. Err.	z	*p*	95% CI Lower	95% CI Upper
Subregion x age^2						
Presubic.	-0.0453376	0.0209391	-2.17	0.03	-0.0863774	-0.0042978
Subiculum	-0.087531	0.020729	-4.22	0.0001	-0.1281591	-0.0469029
CA1	-0.0704198	0.0288827	-2.44	0.015	-0.1270288	-0.0138109
CA3	-0.0156939	0.014228	-1.1	0.27	-0.0435803	0.0121925
CA4	-0.0222503	0.0121539	-1.83	0.067	-0.0460714	0.0015708
Mol. layer	-0.0697179	0.0204835	-3.4	0.001	-0.1098648	-0.029571
HATA	-0.0027531	0.0162132	-0.17	0.865	-0.0345303	0.0290241
Fimbria	-0.0308448	0.0191435	-1.61	0.107	-0.0683655	0.0066758
GC-DG	-0.0308096	0.0125874	-2.45	0.014	-0.0554805	-0.0061387
Hipp. tail	-0.1160412	0.0295856	-3.92	0.0001	-0.174028	-0.0580544
Total grey matter vol.	0.0003162	0.0000243	13.02	0.0001	0.0002686	0.0003638
Sex (ref: male)	0.4466621	2.272799	0.2	0.844	-4.007942	4.901266
Education (ref: primary/none)						
Secondary	-3.642995	2.44173	-1.49	0.136	-8.428698	1.142708
Third/higher	-4.693827	2.493382	-1.88	0.06	-9.580765	0.1931112
Current/past smoker (ref: never smoked)	1.799415	1.807804	1.0	0.32	-1.743816	5.342645
Handedness (ref: right)	-2.308146	3.207078	-0.72	0.472	-8.593904	3.977613
Cardiac disease (ref: none)	-5.880229	2.525205	-2.33	0.02	-10.82954	-0.9309189
Intercept	-121.5923	84.43171	-1.44	0.15	-287.0755	43.89077
Random effects parameters						
Participant var	220.0317	24.23295	N/A	N/A	177.3124	273.0432
Hemisphere var	234.5004	16.21626	N/A	N/A	204.7769	268.5382
Residuals var						
Presubic.	1230.247	59.38431	N/A	N/A	1119.192	1352.322
Subiculum	1193.238	57.76756	N/A	N/A	1085.221	1312.006
Parasubic.	623.5396	29.95679	N/A	N/A	567.549	685.107
CA1	2903.572	139.5029	N/A	N/A	2642.63	3190.28

Term	Estimate	Std. Err.	z	*p*	95% CI Lower	95% CI Upper
CA3	232.3805	11.24457	N/A	N/A	211.3543	255.4983
CA4	1.019755	1.512874	N/A	N/A	0.0556778	18.6771
Mol. layer	1150.456	55.55286	N/A	N/A	1046.568	1264.656
HATA	487.8853	23.5053	N/A	N/A	443.9239	536.2
Fimbria	925.9533	44.73891	N/A	N/A	842.2906	1017.926
GC-DG	46.37387	2.366836	N/A	N/A	41.95944	51.25274
Hipp. tail	3077.355	147.4349	N/A	N/A	2801.54	3380.324

Supplementary table 3: Animal naming (AN) fully adjusted model output

Term	Estimate	Std. Err.	z	*p*	95% CI Lower	95% CI Upper
Subregion (ref: Parasubic.)						
Presubic.	123.0444	74.69081	1.65	0.099	-23.34692	269.4357
Subiculum	67.48078	90.11154	0.75	0.454	-109.1346	244.0961
CA1	368.1414	149.7879	2.46	0.014	74.56251	661.7202
CA3	123.2055	64.811	1.9	0.057	-3.821685	250.2328
CA4	148.8614	49.60835	3.0	0.003	51.63083	246.092
Mol. layer	348.0783	107.8584	3.23	0.001	136.6797	559.4768
HATA	0.3772842	31.83129	0.01	0.991	-62.0109	62.76547
Fimbria	-71.04233	48.67718	-1.46	0.144	-166.4478	24.36318
GC-DG	178.6825	57.66097	3.1	0.002	65.66912	291.696
Hipp. tail	134.2273	140.9655	0.95	0.341	-142.0599	410.5145
AN	0.0415445	0.1094801	0.38	0.704	-0.1730325	0.2561216
Subregion x AN						
Presubic.	0.561801	0.2209305	2.54	0.011	0.1287852	0.9948168
Subiculum	0.0066676	0.266544	0.03	0.98	-0.515749	0.5290842
CA1	0.1477009	0.4430627	0.33	0.739	-0.720686	1.016088
CA3	0.0893117	0.1917067	0.47	0.641	-0.2864265	0.4650498
CA4	0.1934462	0.1467382	1.32	0.187	-0.0941555	0.4810478
Mol. layer	0.2698257	0.319038	0.85	0.398	-0.3554773	0.8951286
HATA	0.0330235	0.0941549	0.35	0.726	-0.1515166	0.2175636
Fimbria	0.1164187	0.1439839	0.81	0.419	-0.1657845	0.3986219
GC-DG	0.209401	0.1705573	1.23	0.22	-0.1248852	0.5436873
Hipp. tail	0.5564656	0.4169665	1.33	0.182	-0.2607738	1.373705
Age	-0.2796054	1.053981	-0.27	0.791	-2.345371	1.78616
Age^2	0.0044625	0.0076371	0.58	0.559	-0.010506	0.019431
Subregion x age						
Presubic.	4.487421	2.168075	2.07	0.038	0.2380714	8.736771
Subiculum	10.42076	2.615698	3.98	0.00001	5.294082	15.54743

Term	Estimate	Std. Err.	z	*p*	95% CI Lower	95% CI Upper
CA1	7.997668	4.347943	1.84	0.066	-0.5241442	16.51948
CA3	1.391027	1.881291	0.74	0.46	-2.296235	5.078289
CA4	2.020735	1.439998	1.4	0.161	-0.80161	4.843079
Mol. layer	6.915266	3.130842	2.21	0.027	0.7789295	13.0516
HATA	0.2981528	0.9239776	0.32	0.747	-1.51281	2.109116
Fimbria	3.514242	1.412969	2.49	0.013	0.7448739	6.28361
GC-DG	2.802074	1.673744	1.67	0.094	-0.478405	6.082552
Hipp. tail	12.1354	4.091852	2.97	0.003	4.115516	20.15528
Subregion x age^2						
Presubic.	-0.0453182	0.0156899	-2.89	0.004	-0.0760698	-0.0145666
Subiculum	-0.0915362	0.0189292	-4.84	0.00001	-0.1286369	-0.0544356
CA1	-0.0775817	0.0314651	-2.47	0.014	-0.1392522	-0.0159112
CA3	-0.0165555	0.0136145	-1.22	0.224	-0.0432394	0.0101284
CA4	-0.0235354	0.010421	-2.26	0.024	-0.0439601	-0.0031108
Mol. layer	-0.0736655	0.0226572	-3.25	0.001	-0.1180728	-0.0292581
HATA	-0.0046306	0.0066866	-0.69	0.489	-0.0177362	0.0084749
Fimbria	-0.0322839	0.0102253	-3.16	0.002	-0.0523252	-0.0122425
GC-DG	-0.0326399	0.0121125	-2.69	0.007	-0.05638	-0.0088998
Hipp. tail	-0.1152019	0.0296118	-3.89	0.00001	-0.1732401	-0.0571638
Total grey matter vol.	0.0001579	0.0000115	13.68	0.00001	0.0001353	0.0001805
Sex (ref: male)	-0.6457086	1.060777	-0.61	0.543	-2.724794	1.433376
Education (ref: primary/none)						
Secondary	-2.739217	1.164294	-2.35	0.019	-5.021191	-0.457243
Third/higher	-2.635385	1.194126	-2.21	0.027	-4.975829	-0.2949403
Current/past smoker (ref: never smoked)	0.0070375	0.8549602	0.01	0.993	-1.668654	1.682729
Handedness (ref: right)	-1.799715	1.532505	-1.17	0.24	-4.803369	1.203939
Cardiac disease (ref: none)	-2.822095	1.188532	-2.37	0.018	-5.151574	-0.4926151
Intercept	-30.11325	36.9652	-0.81	0.415	-102.5637	42.33721

Term	Estimate	Std. Err.	z	*p*	95% CI Lower	95% CI Upper
Random effects parameters						
Participant var	55.96477	5.91719	N/A	N/A	45.49007	68.8514
Hemisphere var	10.69511	2.748748	N/A	N/A	6.462761	17.69915
Residuals var						
Presubic.	931.7526	46.36625	N/A	N/A	845.1675	1027.208
Subiculum	1412.923	70.57024	N/A	N/A	1281.162	1558.234
Parasubic.	124.4929	7.71931	N/A	N/A	110.2465	140.5802
CA1	4123.501	201.3206	N/A	N/A	3747.21	4537.578
CA3	670.8015	34.16381	N/A	N/A	607.0752	741.2174
CA4	341.4578	19.11082	N/A	N/A	305.9826	381.046
Mol. layer	2078.121	103.7431	N/A	N/A	1884.419	2291.733
HATA	67.34705	5.563455	N/A	N/A	57.27985	79.18361
Fimbria	324.1298	16.93821	N/A	N/A	292.5751	359.0878
GC-DG	505.0051	27.36972	N/A	N/A	454.1123	561.6015
Hipp. tail	3637.828	176.764	N/A	N/A	3307.363	4001.313

Supplementary table 4: test-retest reliability data for immediate recall (IR), delayed recall (DR) and animal naming (AN) tasks in TILDA MRI cohort, across Waves 1-3.

	IR1 W1	IR2 W1	DR W1	IR1 W2	IR2 W2	DR W2	IR1 W3	IR2 W3	DR W3	AN W1	AN W2	AN W3
IR1 W1	1											
IR2 W1	0.62	1										
DR W1	0.54	0.65	1									
IR1 W2	0.44	0.43	0.42	1								
IR2 W2	0.38	0.47	0.48	0.68	1							
DR W2	0.38	0.46	0.53	0.65	0.70	1						
IR1 W3	0.35	0.37	0.38	0.44	0.41	0.37	1					
IR2 W3	0.35	0.39	0.41	0.43	0.44	0.43	0.67	1				
DR W3	0.37	0.41	0.51	0.43	0.43	0.53	0.59	0.72	1			
AN W1	0.27	0.32	0.32	0.20	0.24	0.24	0.17	0.21	0.20	1		
AN W2	0.22	0.28	0.31	0.35	0.39	0.44	0.24	0.24	0.24	0.49	1	
AN W3	0.20	0.20	0.23	0.25	0.25	0.27	0.26	0.34	0.28	0.44	0.50	1

N=513. IR1: immediate recall attempt 1; IR2: immediate recall attempt 2; DR: delayed recall; AN: animal naming; W1: Wave 1; W2: Wave 2; W3: Wave 3.

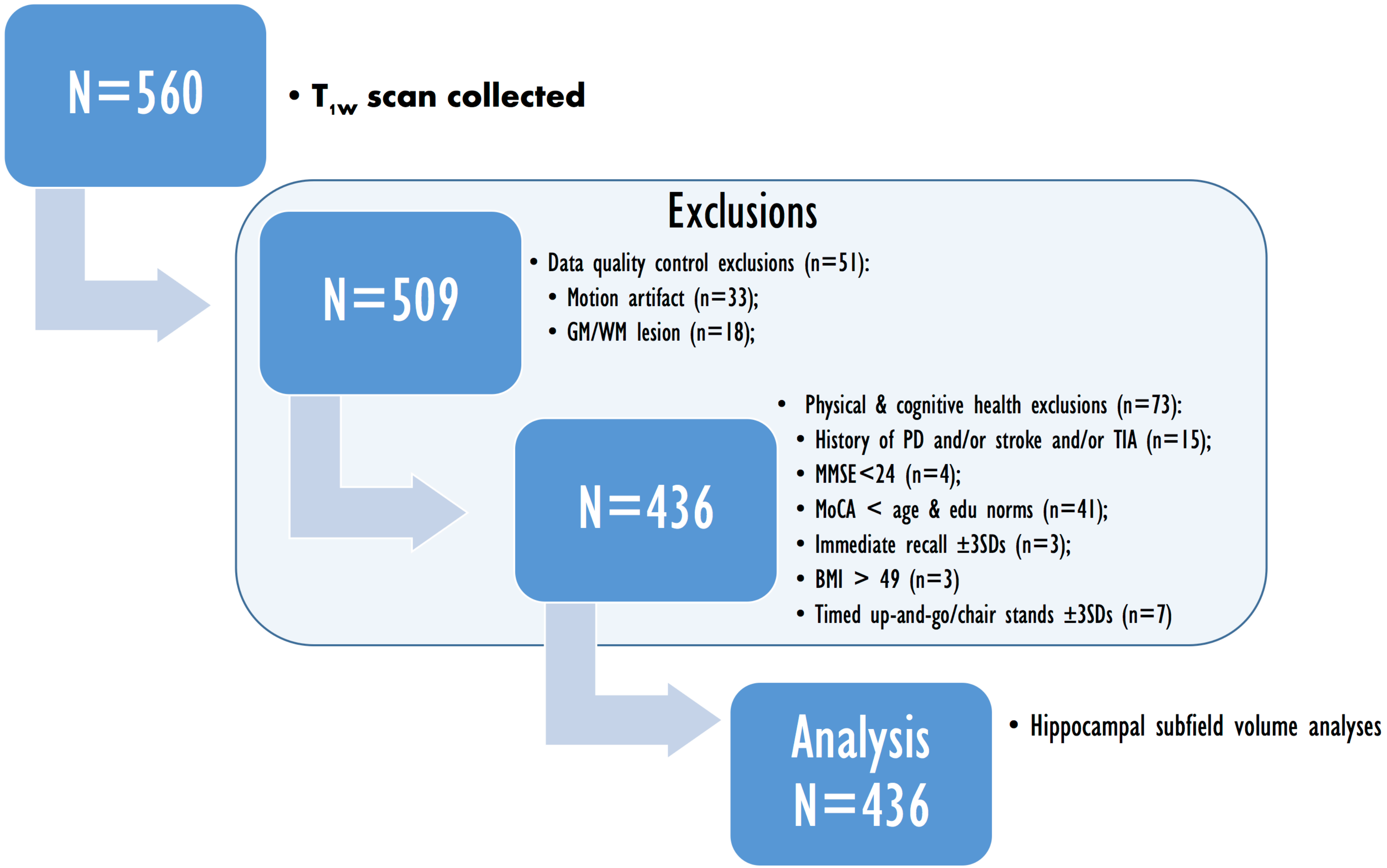
N=560
• T1w scan collected
Exclusions
N=509
• Data quality control exclusions (n=51):
• Motion artifact (n=33);
• GM/WM lesion (n=18);
• Physical & cognitive health exclusions (n=73):
• History of PD and/or stroke and/or TIA (n=15);
• MMSE<24 (n=4);
• MoCA < age & edu norms (n=41);
• Immediate recall ±3SDs (n=3);
• BMI > 49 (n=3)
• Timed up-and-go/chair stands ±3SDs (n=7)
N=436
Analysis
N=436
• Hippocampal subfield volume analyses

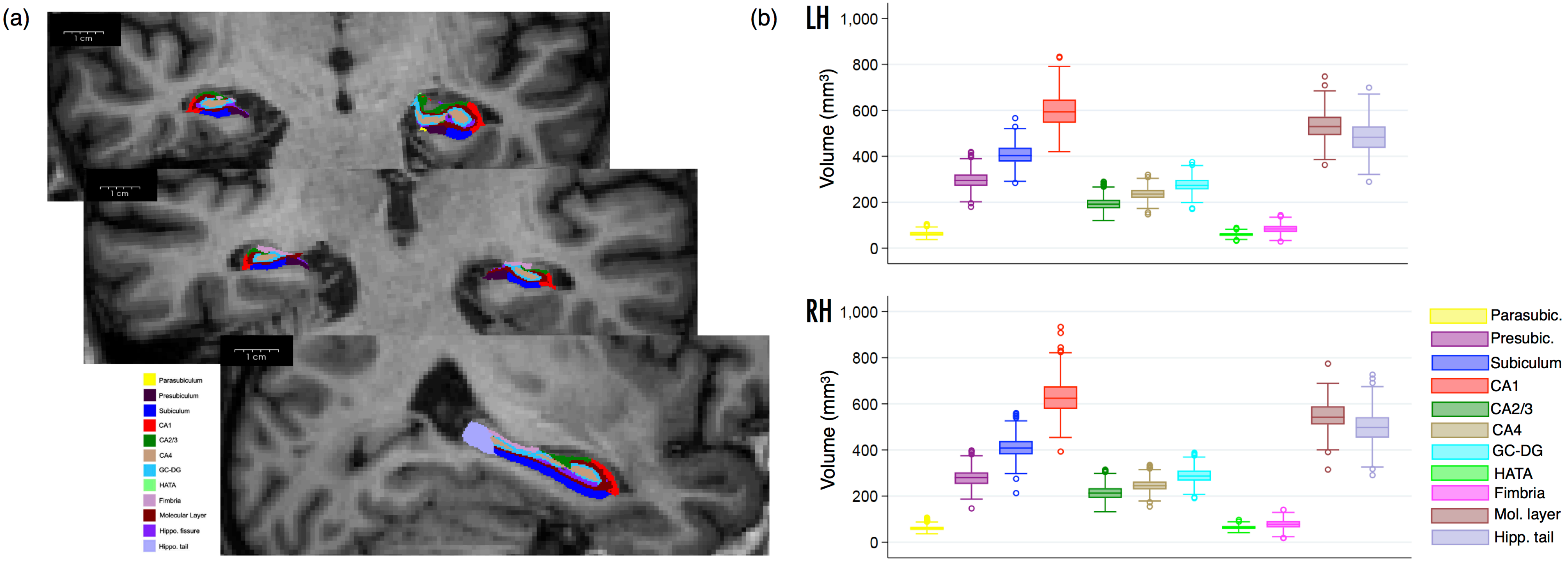
(a)
1 cm
1 cm
1 cm
Parasubiculum
Presubiculum
Subiculum
CA1
CA2/3
CA4
GC-DG
HATA
Fimbria
Molecular Layer
Hippo. fissure
Hippo. tail
(b)
LH
RH
Volume (mm³)
1,000
800
600
400
200
0
Parasubic.
Presubic.
Subiculum
CA1
CA2/3
CA4
GC-DG
HATA
Fimbria
Mol. layer
Hipp. tail

Supplementary Figure S1: Exclusionary criteria leading to analysis sample for hippocampal subfield volumes, N=436. PD - Parkinson's disease; TIA - transient ischemic attack; MMSE - Mini-mental State Examination; MoCA - Montreal Cognitive Assessment. Timed up-and-go: time (s) taken to rise from chair, walk 3m, walk back to chair, and return to seated position. Chair stands: time taken to repeat five stands from seated position, without assistance of arms. MoCA age and education norms based on [9].

Supplementary Figure S2: Hippocampal subfield parcellation summary. (a) Two coronal views and one sagittal view of hippocampal subfield parcellations, for a representative TILDA participant; subfields colour coded (see legend, left inset). (b) Box plots of hippocampal subfield volumes (mm^3) for left and right hemispheres for the analysis sample, N=436; subfields colour coded (see legend, right inset).
